# Characterization of Ambrette Seed Oil and Its Mode of Action in Bacteria

**DOI:** 10.3390/molecules20010384

**Published:** 2014-12-29

**Authors:** Selvaraj Arokiyaraj, Seong Ho Choi, Yoonseok Lee, Rajaraman Bharanidharan, Villianur Ibrahim Hairul-Islam, Badathala Vijayakumar, Young Kyoon Oh, Vannam Dinesh-Kumar, Savariar Vincent, Kyoung Hoon Kim

**Affiliations:** 1 Institute of Green Bio Science & Technology, Seoul National University, Pyeongchang-daero 232916, Korea; E-Mails: arokiyaraj16@gmail.com (S.A.); yoonseok95@snu.ac.kr (Y.L.); 2 Department of Animal Science, Chungbuk National University, Chunbuk 361763, Korea; E-Mail: seongho@cbnu.ac.kr; 3 Department of Biotechnology, Vel Tech High Tech Dr.Rangarajan Dr.Sakunthala Engineering College (Anna University), Chennai 600062, India; E-Mail: bharanitharshan76@gmail.com; 4 Pondicherry Centre for Biological Sciences, Pondicherry 605005, India; E-Mail: pcbsresearch@gmail.com; 5 Department of Chemistry, Vel Tech High Tech Dr.Rangarajan Dr.Sakunthala Engineering College (Anna University), Chennai 600062, India; E-Mail: badathala@rediffmail.com; 6 Department of Animal Nutrition and Physiology, National Institute of Animal Science, Rural Development Administration, Suwon 441706, Korea; E-Mail: oh665@korea.kr; 7 Centre for Environment Research and Development, Loyola College, Chennai 600034, India; E-Mails: zoomin.dinesh@gmail.com (V.D.-K.); svincentloyola@gmail.com (S.V.); 8 Graduate School of International Agricultural Technology, Seoul National University, Pyeongchang 232916, Korea

**Keywords:** ambrette seed oil, antibacterial activity, chemical composition, docking, DHFR

## Abstract

In the present study, chemical composition and the antibacterial mechanism of ambrette seed oil are investigated. Chemical composition of the oil was analysed by gas chromatography-mass spectrometry (GC-MS). Thirty-five compounds were identified and the major compounds were found to be farnesol acetate (51.45%) and ambrettolide (12.96%). The antibacterial activity was performed by well diffusion assay and the mechanisms were studied by measuring the alkaline phosphatase (ALP), lactate dehydrogenase (LDH) and protein leakage assays. The antibacterial effect of the ambrette seed oil showed inhibitory effect against *Bacillus subtilis*, *Staphylococcus aureus* and *Enterococcus faecalis*. The LDH activity was high in all tested bacteria compared with control, whereas the ALP and protein concentrations were also increased in *E. faecalis*. Molecular docking revealed the ligands farnesol acetate and ambrettolide had satisfactory binding energy towards the beta lactamase TEM-72 and dihydrofolate reductase (DHFR) protein. Due to its better antibacterial properties, the ambrette seed oil could be used as a source of antibacterial agents.

## 1. Introduction

The development of drug resistance by bacteria is a worldwide problem. These bacteria cross world boundaries with ease, and world health leaders have described the drug resistance by bacteria as a “nightmare” that poses “catastrophic” threat to people in every country [1]. Several investigations have dealt with the problem of antibiotic resistance in *P. aeruginosa*, *E. faecalis* and *K. pneumoniae* [2,3]. Though pharmaceutical companies have developed an avalanche of novel antibiotics in recent years, resistance to them by microorganisms has increased. Thus, it has necessitated the search for safe and cost effective compounds for the treatment of bacterial infections.

For a long period of time, plant products have been used for various medicinal purposes. The composition and antibacterial activity of essential oils from medicinal plants and their secondary metabolites have been widely studied and reviewed [4,5,6,7]. Therefore, secondary metabolites from plant oils and extracts have become attractive in many pharmaceutical and food processing industries [8].

*Abelmoschus moschatus* L. (Malvaceae) is an evergreen shrub that is also known as Muskdana. It has been growing throughout India and cultivated in China, West Indies and Indonesia. The seeds are used for various therapeutic purposes which include treating headaches, cramps, muscular aches and pains, depression and other nervous complaints [9]. A brief overview of the traditional medicinal use of *A. moschatus* indicates its effectiveness in the treatment of various bacteriological pathogenesis [10,11]. However, beyond aqueous and ethanolic extracts of *A. moschatus* (leaf and seed), there are no reports for the antimicrobial activity of ambrette seed oil to the best of our knowledge. The present study determines the chemical composition of oil derived from the seeds of *A. moschatus*, screens *in vitro* antibacterial activity of ambrette seed oil against bacteria and reports the mechanism of action for the first time.

## 2. Results and Discussion

### 2.1. Identification of Bioactive Compounds

GC-MS analysis of ambrette seed oil reveals 35 compounds. All these constituents were identified and characterized by comparison with the data available in NIST library (Table 1). The major components of the oil were farnesol acetate (51.45%) and ambrettolide (12.96%). These two compounds have been selected for molecular docking study with extended-spectrum β-lactamases TEM-72 and DHFR (dihydrofolate reductase) proteins for their possible antibacterial action. Previous report evidenced that *A. moschatus* seed oil has 2E,6E–farnesyl acetate (59.1%), hexadec-7-en-16-olide (7.8%), decyl acetate (5.6%) and volatile compounds [12,13,14]. Farnesol acetate is an ester of farnesol and acetic acid widely distributed in many essential oils such as citronella, lemon grass, musk and badam (almond).

**Table 1:** Chemical composition of ambrette seed oil.

Peak No	Components	Class of Compound	Retention Time	SI	Area %
1	Butanoicacid,octyl ester	Lipid	26.37	816	0.24
2	Acetic acid, decyl ester	Lipid	27.04	961	6.53
3	Ethanone, 1,1'-(1,3-phenylene)bis-	Aromatic diketone	27.71	838	0.18
4	Butanoic acid, 2-methyl-, octyl ester	Lipid	38.68	758	0.15
5	5,9-Undecadien-2-one, 6,10-dimethyl-	Lipid	28.37	891	0.29
6	Cis-a-Farnesene	Sesquiterpene	28.51	857	0.36
7	Aromandendrene	Sesquiterpene	28.72	833	0.10
8	1-Dodecanol	Lipid	29.00	856	0.23
9	Phenol, 2,5-bis(1-methylethyl)-	Phenol	29.07	724	0.12
10	Bicyclo[3.1.1]hept-2-ene, 2,6-dimethyl-6(4-methyl-3-pentenyl)-	Sesquiterpene	29.68	828	0.17
11	E-10-Dodecen-1-ol propionate	Lipid	29.86	779	0.34
12	a-Farnesene	Sesquiterpene	30.09	881	0.86
13	Phenol, 3,5-bis(1-methylethyl)-	Phenol	30.19	824	0.09
14	1,6,10-Dodecatrien-1-ol,3,7,11-trimethyl-, (E)-	Sesquiterpene	31.72	906	2.66
15	5-Dodecen-1-ol, acetate, (Z)-	Lipid	32.45	931	2.09
16	Lauryl acetate	Lipid	32.02	913	7.80
17	(Z)6-Pentadecen-1-ol	Lipid	35.37	840	0.22
18	9,12-Octadecadienoyl chloride, (Z,Z)-	Lipid (acid chloride)	35.53	771	0.42
19	Nerolidyl acetate	Sesquiterpene	35.94	812	0.30
20	17-Octadecynoic acid	Lipid	36.79	792	0.16
21	9,12-Tetradecadien-1-ol, acetate, (Z,E)	Lipid	37.58	848	0.67
22	5-Tetradecen-1-ol, acetate (Z)-	Lipid	37.80	942	3.74
23	8-Hexadecenal, 14-methyl-(Z)-	Lipid	38.40	783	0.53
24	Pentacyclo [9.1.0(2,4).0.(5,7).8(8,10)]dodecane	Lipid (Cyclic)	38.75	797	0.86
25	**Farnesol acetate**	Sesquiterpene	39.37	947	51.45
26	a-Guaiene	Sesquiterpene	39.55	759	1.16
27	1,3,6,10-Cyclotetradecatetraene, 3,7,11-trimethyl-14-(1-methylethyl)	Sesquiterpene	40.97	833	0.31
28	Cyclopropanebutanoic acid	Lipid	41.30	780	0.11
29	Propanoic acid, 2,2-dimethyl	Lipid	41.43	842	1.45
30	9-Hexadecenoic acid	Lipid	41.55	817	0.10
31	**Ambrettolide**	Lactone	41.78	848	12.96
32	Caryophyllene oxide	Oxygenated Sesquiterpene	42.11	824	0.37
33	5,8,11,14-Eicosatetraenoic acid, methyl ester	Lipid	43.30	807	0.18
34	Nerolidyl acetate	Sesquiterpene	44.44	816	0.56
35	9,12-Octadecadienoic acid	Lipid	46.48	841	2.03

Compound proportions were calculated from the chromatograms obtained on the TG-5MS column. Bold values correspond to the major compounds of the essential oils.

### 2.2. Antibacterial Assay of Ambrette Seed Oil

Antibacterial activity of the ambrette seed oil against Gram positive and Gram negative bacteria are shown in Table 2. The oil showed satisfactory inhibitory effect on *B. subtilis*, *S. aureus* and *E. faecalis* whereas less active against *P. aeruginosa*. The oil possesses farnesol acetate and ambrettolide—the major components—which may be responsible for the antibacterial action. An early report [15] evidenced that farnesol showed significant inhibitory effect against *S. aureus* and our results are in agreement with the previous report. The antimicrobial action of ambrette seed oil may be due to its hydrophobicity, which causes damage in the cell membrane of bacteria and leakage of cellular constituents [16]. It has been evidenced that the Gram positive bacteria are more sensitive to essential oils than Gram negative bacteria due to the presence of a highly charged outer membrane that acts as a protective barrier [17,18]. Similarly the leaf and seed extract of *A. moschatus* showed antibacterial effect against *B. subtilis* and *S*. *aureus* [10].

**Table 2:** Antibacterial activity of ambrette seed oil and streptomycin against bacteria in Mueller Hinton agar by well diffusion assay.

Bacterial Strain	Zone of Inhibition (mm)	
	Ambrette Seed Oil (18 mg/mL)	Streptomycin (30 µg/mL)
*B. subtilis*	12 ± 0.5	28 ± 1.2
*S. aureus*	13 ± 0.5	20 ± 1.4
*E. faecalis*	13 ± 0.8	15 ± 1.6
*P. aeruginosa*	09 ± 0.7	15 ± 1.5

Values are expressed in mean ± standard error SE (n = 3).

### 2.3. ALP Quantification Assay

**Figure 1:**
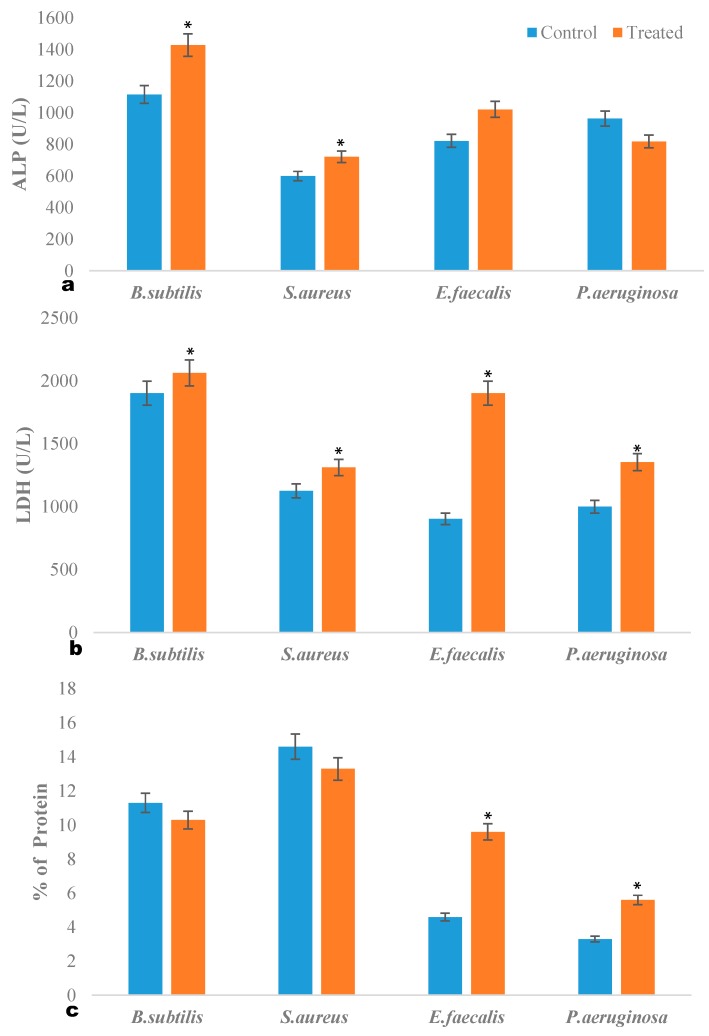
(**a**) ALP quantification assay, (**b**) LDH quantification assay, (**c**) Protein leakage assay. Values are expressed as mean ± standard error (SE). * *p* < 0.05, Experiments performed in triplicates.

Alkaline phosphatase is an enzyme present in the periplasmic space of the bacteria. The enzyme activity increases during phosphate starvation and sporulation [19]. In our study, the ALP levels of *B. subtilis, S. aureus,* and *E. faecalis* were increased upon treatment with ambrette seed oil (Figure 1a). This may be due to the oxidative stress imposed on the bacteria by the essential oil, and in order to overcome the phosphate starvation, bacteria produce higher amounts of ALP. Treatment with oil caused the bacteria to generate oxidative stress by the reactive oxygen species (ROS). High levels of ROS can increase oxidative stress that leads to damage in cell membrane, protein and intra cellular respiratory system [20]. In the present results, *B. subtilis* (*p* < 0.05) and *S. aureus* (*p* < 0.05) produced higher amounts of ALP when compared with gram negative bacteria. Similarly, Chesnut *et al.* (1991) have reported the expression of phoAIII gene of *B. subtilis* which causes expression of ALP during phosphate starvation and sporulation [21]. This study showed the possible stimulation of oxidative stress, from the bacterial cell after exposure to ambrette seed oil.

### 2.4. LDH Quantification Assay

LDH is a cytosolic enzyme in bacteria, measured to determine the stress induced membrane damage caused by ambrette seed oil treatment (Figure 1b). LDH activity was determined by measuring the reduction of NAD^+^ to NADH and H^+^ during the oxidation of lactate to pyruvate. In the present study, there was a significant increase in LDH activity in all tested bacteria (*p* < 0.05) compared with control. The results indicate that ambrette seed oil caused membrane damage to the bacterial cells and resulted in cell death. It has been reported that the cell membrane permeability changes as the plant molecules interact with the microbial cell surface to cause the leakage of the intracellular component and cause a bactericidal effect. Tsai and Su (1999) have reported treatment of *Escherichia coli* with chitosan (800 ppm) which induced the leakage of glucose and LDH into extracellular media of *E. coli* cells [22].

### 2.5. Intracellular Protein Leakage

Exposure to ambrette seed oil showed increase in the protein concentration on *E. faecalis* (*p* < 0.05) and *P. aeruginosa* (*p* < 0.05) compared with control, indicating disruption of the bacterial membrane (Figure 1c). Moreover, the antibacterial activity of the ambrette seed oil against the Gram positive bacteria on *B. subtilis* and *S. aureus* found to be less active. Henie *et al.* (2009) reported similar bactericidal effect on food pathogens by *Psidium guajava* leaf extracts [23].

### 2.6. In Silico Studies

**Figure 2:**
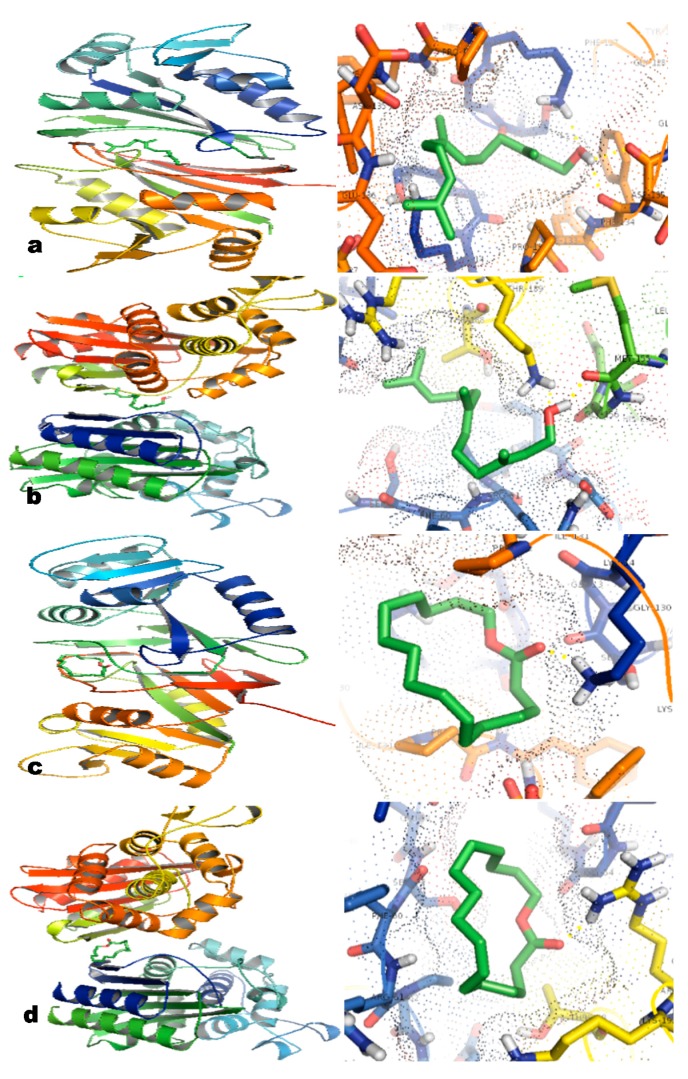
Putative binding pose of ligands docked with DHFR and TEM-72. The yellow dotted line indicates the H-bonding between the ligand and protein. (**a**) Molecular interaction of ligand farnesol acetate with DHFR. (**b**) Molecular interaction of ligand farnesol acetate with TEM-72. (**c**) Molecular interaction of ligand ambrettolide with DHFR. (**d**) Molecular interaction of ligand of ambrettolide with TEM-72.

The two major compounds were docked with the target proteins. After docking, optimal interaction and best affinity score was used to interpret the best conformation among the generated conformations for each ligand. The two ligands showed satisfactory binding towards the proteins DHFR and TEM-72 protein. Farnesol acetate and ambrettolide have excellent energy values of −5.95 Kcal·mol^−1^ and −5.67 Kcal·mol^−1^ towards DHFR, respectively. Both compounds also exhibited fair binding towards TEM-72 with moderate energy values of −4.47 Kcal·mol^−1^ and −3.77 Kcal·mol^−1^, respectively. It is observed that the lower the energy value the better the ligand is docked to the receptor. Hydrogen (H) bonding plays an important role in determining the structure and function of any biological molecule, especially for its inhibition in a complex [24]. In this study, the ligand farnesol acetate was stabilized by two H-bonds with B:PHE134:O and A:LYS14:HZ1 residues of DHFR (Figure 2a) and A:LYS:152 B:METH:195 residues of TEM-72 (Figure 2b). Similarly, ambrettolide was stabilized by a single H-bond with A:LYS14:HZ3 residue of the DHFR (Figure 2c) and B:ARG:191 residue of TEM-72 (Figure 2d). This computational study suggests that the major compounds of the ambrette seed oil integrated with the beta lactam protein TEM-72 and DHFR protein, which are responsible for antibiotic resistance and reduce the activity of proteins, thereby making the microbe sensitive to drugs.

## 3. Experimental Section

The ambrette seed oil was obtained from Sigma–Aldrich (cat. No. W205001, Saint Louis, MO, USA). All of the media and chemicals were purchased from DIFCO (Detroit, MI, USA) and Sigma chemicals (Saint Louis, MO, USA).

### 3.1. GC-MS Analysis

The ambrette seed oil was analyzed by a Thermo Trace 1310 (Gas chromatograph) system with a Thermo ISQ LT mass selective detector. The machine was equipped with a TG-5MS (Mass spectroscopy) column (30 × 0.25 mm (5%-phenyl)–methylpolysiloxane capillary column, film thickness ×0.25 µm), temperature of the injector was 220 °C and temperature of the transfer line was 250 °C. The oven temperature was programmed as follows: initial temperature; 50 °C for 5 min, and then raised 4 °C/min up to 250 °C. The carrier gas was Helium. The amount of sample injected was 1 µL (split ratio 1:10) and the ionization energy was 70 eV. Identification of oil components was based on their retention time and by comparison of their mass spectral pattern with the available data in MS library (NIST MS Search Program V. 2.0 g).

### 3.2. Microorganisms

*Bacillus subtilis* (ATCC 9372), *Staphylococcus aureus* (ATCC 25923), *Enterococcus faecalis* (ATCC 29212), and *Pseudomonas aeruginosa* (ATCC 27853) were obtained from the Korean Culture Centre of Microorganisms, Seoul, Korea. All bacterial strains were maintained at MHA slants and stocks were stored at −20 °C until use.

### 3.3. Antibacterial Assay

The antibacterial efficacy was assayed by the agar well diffusion method against Gram-positive and Gram-negative bacteria [25]. The cultures were sub cultured on Muller–Hinton broth at 37 °C on a rotary shaker at 200 rpm and individual bacterial strain was swabbed on the petri plates using sterile cotton swabs. Wells of size 6 mm were made on Muller–Hinton agar plates using gel puncture. Twenty μL of ambrette seed oil at the concentration of 18 mg/mL was suspended in 0.02% Tween 80 [26], seeded into the wells and incubated at 37 °C for 18 h. Tween 80 was used to improve the diffusion rate of oil in aqueous solid and liquid media. Streptomycin (30 µg) was used as a control. The zone of inhibition in the diameter of each well was measured. The experiments were performed in three replicates.

### 3.4. ALP Quantification (Alkaline Phosphatase)

ALP was measured in oil treated culture supernatants using an ALP assay kit (Linear Chemicals, Montgat, Barcelona, Spain) which involves a colorimetric determination of *p*-nitrophenol released at 405 nm. Cells were cultured in nutrient broth treated with 18 mg/mL of ambrette seed oil. After 24 h of incubation, cell free supernatants were collected. All the treatments were compared against control wells (cells without treatment) and the final results were expressed in units/liter.

### 3.5. Lactate Dehydrogenase Quantification (LDH)

The bacterial cells were first incubated with 18 mg/mL of ambrette seed oil for 24 h at 37 °C. After incubation, 50 μL of the upper medium were collected from each well. The untreated cells were then lysed with a cell lysis solution for 40 min at room temperature and the lysate was collected. LDH activity was measured using LDH release quantification cytotoxicity assay kit (Thermo Scientific, Rockford, IL, USA), in accordance with manufacturer’s instructions. All the treatments were compared against control wells (cells without treatment) and the final results were expressed in units/liter.

### 3.6. Protein Leakage

The bacterial cultures were treated with 18 mg/mL of the oil extract from ambrette seed and incubated for 24 h at 37 °C. After incubation the cells were centrifuged at 5000 rpm for 10 min and the supernatants were collected. To determine the protein level in the supernatant of treated and untreated pathogens the assay was carried out according to the method of Bradford (1976) [27]. The supernatant was added with 2 mL of 0.5 M NaOH and 0.1 mL of 0.1 N folin phenol reagent; the absorbance was read at 550 nm after 10 min. The percentage of protein released from the cells was determined using the units/liter of protein.

### 3.7. Ligand and Target Protein Preparation

The 3D structure of major compounds such as farnesol acetate and ambrettolide have been retrieved as PDB file from the PUBCHEM database. Dihydrofolate reductase (DHFR) a macromolecule enzyme which is necessary for biosynthesis in bacteria and TEM-72, a class A β-lactamases enzyme represent bacterial resistant factors against β–lactam antibiotics were selected as target molecules [28,29]. The 3D structure of TEM-72 and DHFR available at the Protein Data Bank were retrieved in PDB format [30]. To study the nature of the interactions of the major molecules with the target protein, docking was carried out using AUTODOCK4.0. For the protein structure, polar hydrogen atoms, Kolmann charges and Gagstier charges were added and the structures were then saved in PDBQT file format for docking. For the ligand molecule, the roots were detected and an energy grid was built within a cubic box of dimensions 60 × 60 × 60 Å grid points and 0.375 Å spacing using the Autogrid program. Molecular docking was performed based on Lamarckian Genetic Algorithm. Best run coordinates of the drug(s) with enzymes were analyzed and visualized through python molecule viewer (PMV) for analysis of their modes of interaction with binding site residues [31].

### 3.8. Statistical Analysis

All experiments were done in triplicate, and then the values were expressed as the mean ± SE. Statistical significance was evaluated by Student’s *t*-test (*p <* 0.05), using the Statistical Package for the Social Sciences (SPSS version 21; SPSS Inc., Chicago, IL, USA).

## 4. Conclusions

The ambrette seed oil showed satisfactory activity against *B. subtilis*, *S. aureus* and *E. faecalis* probably by interfering with the cell membrane and by the generation of reactive oxygen species. Further studies are required in isolation, and characterization of the active compound responsible for the antibacterial effect.
